# Anti-tumor effect of dandelion flavone on multiple myeloma cells and its mechanism

**DOI:** 10.1007/s12672-024-01076-z

**Published:** 2024-06-08

**Authors:** Hua Gui, Xiaohong Fan

**Affiliations:** Present Address: Hematology Department, QingPu Branch of ZhongShan Hospital Affiliated to Fudan University, 1158 Park Road(E), Qingpu, Shanghai, China

**Keywords:** Dandelion flavone, Traditional Chinese medicine, Multiple myeloma, Macrophages, PI3K/AKT pathway

## Abstract

**Background:**

Multiple myeloma (MM) is a prevalent hematologic malignancy characterized by the uncontrolled proliferation of monoclonal plasma cells in the bone marrow and excessive monoclonal immunoglobulin production, leading to organ damage. Despite therapeutic advancements, recurrence and drug resistance remain significant challenges.

**Objective:**

This study investigates the effects of dandelion flavone (DF) on MM cell proliferation, migration, and invasion, aiming to elucidate the mechanisms involved in MM metastasis and to explore the potential of traditional Chinese medicine in MM therapy.

**Methods:**

DF's impact on myeloma cell viability was evaluated using the CCK-8 and colony formation assays. Cell mobility and invasiveness were assessed through wound healing and transwell assays, respectively. RT-PCR was employed to quantify mRNA levels of MMP-2, MMP-9, TIMP-1, and TIMP-2. Apoptotic rates and molecular markers were analyzed via flow cytometry and RT-PCR. The PI3K/AKT signaling pathway was studied using Western blot and ELISA, with IGF-1 and the PI3K inhibitor LY294002 used to validate the findings.

**Results:**

DF demonstrated dose-dependent inhibitory effects on MM cell proliferation, migration, and invasion. It reduced mRNA levels of MMP-2 and MMP-9 while increasing those of TIMP-1 and TIMP-2. Furthermore, DF enhanced the expression of pro-apoptotic proteins and inhibited M2 macrophage polarization by targeting key molecules and enzymes. The anti-myeloma activity of DF was mediated through the inhibition of the PI3K/AKT pathway, as evidenced by diminished phosphorylation and differential effects in the presence of IGF-1 and LY294002.

**Conclusion:**

By modulating the PI3K/AKT pathway, DF effectively inhibits MM cell proliferation, migration, and invasion, and induces apoptosis, establishing a novel therapeutic strategy for MM based on traditional Chinese medicine.

**Supplementary Information:**

The online version contains supplementary material available at 10.1007/s12672-024-01076-z.

## Introduction

MM is a cancerous carcinoma stemming from B cells. It is marked by the clonal proliferation of plasma cells in the BM and the secretion of monoclonal immunoglobulin or its fragments (M protein). This leads to a range of symptoms such as anemia, abnormal renal function, and bone destruction [1]. This condition is more prevalent among the elderly, and its incidence is steadily rising annually. It is the second most common hematological malignancy, posing a significant threat to human health [2]. The prognosis for patients with MM has been greatly enhanced as a result of the increasing utilization of new drugs. However, some patients experience disease progression and recurrence as a result of drug resistance, ultimately leading to treatment failure [3]. Currently, it is commonly accepted that the BM microenvironment, which offers protection and assistance for the growth of MM cells, plays a crucial role in the emergence of drug resistance and disease progression in MM [4]. Therefore, there is an urgent need to delve into the interactions between MM cells and the BM microenvironment, as well as identify the key elements involved in the interaction.

The interaction between multiple myeloma (MM) cells and the bone marrow (BM) microenvironment is complex and dynamic, affecting their growth, proliferation, drug resistance, angiogenesis, and bone destruction [5]. Adhesion between various cellular components in the BM and MM cells can initiate signal transmission related to proliferation and anti-apoptosis. This interaction also leads to increased expression of cytokines like IL-6, B-cell activating factor (BAFF), and insulin-like growth factors-1 (IGF-1), which promote MM cell growth [6, 7]. These cytokines enhance cell adhesion, stimulate autocrine secretion in MM cells, and trigger paracrine secretion within the BM microenvironment, creating a vicious cycle that drives the disease’s progression. Macrophages (MΦs) are abundant in the BM of MM patients, accounting for about 10% of the total BM stromal cell population, significantly more than other immune cells [8]. Furthermore, MΦs can induce drug resistance in MM cells through interactions involving surface molecular pairs, serving as a prognostic factor in MM [9, 10].

MΦs can be classified into two separate subtypes in accordance with their characteristics and roles: classically activated M1-type and alternative activated M2-type [11]. M1-type MΦs exhibit high expression of IL-12, NO, reactive oxygen species, and MHC, which contribute to the stimulation of T cells and natural killer (NK) cells’ proliferation, enhancing their anti-tumor activities. Conversely, M2-type MΦs display elevated levels of immunosuppressive factors like IL-10 and TGF-β, which inhibit T cell role, promoting immunosuppression and tumor growth [12]. Studies indicate that within the BM microenvironment of MM, MΦs primarily adopt the M2-type phenotype, and their abundance has a tendency to rise as the disease progresses [13]. All these studies suggest that regulating the function of MΦs has great potential to enhance MM treatment efficacy. Therefore, it is crucial to identify the key molecules and mechanisms that regulate the phenotype and function of MΦs in the MM BM microenvironment.

Recent advancements in molecular targeted therapy have made it a central approach in treating malignant tumors, offering precise targeting of cancer cells with reduced toxicity compared to traditional chemotherapy. However, only a subset of patients benefits from this approach [14]. In contrast, traditional Chinese medicine (TCM), with its deep roots in Chinese healing practices, plays a crucial role in cancer treatment. According to TCM, cancer is often seen as a result of qi deficiency and imbalances in Yin and Yang. Treatment strategies in TCM include acupuncture, massage, dietary adjustments, oral medications, and injections [15, 16]. These methods not only help combat cancer but also mitigate the side effects of conventional cancer treatments. Increasingly, the anti-tumor effects of Chinese herbal medicines are gaining attention. Clinical trials have shown that integrating TCM with modern treatment methods leads to improved outcomes, reducing complications, recurrence, metastasis, and enhancing patient quality of life [17, 18].

Dandelion, a perennial herb belonging to the chrysanthemum family, has a long history of medicinal use due to its abundant availability and cost-effectiveness. Dandelion is known to contain dandelion sterols, dandelion alcohol, flavonoids, polysaccharides and other beneficial components. Research on the constituents of dandelion has been on the rise in recent years. Studies conducted both domestically and internationally have demonstrated that the significant effects of dandelion, including suppressing tumor cell proliferation, triggering tumor cell apoptosis, enhancing immunity, and exerting anti-inflammatory properties. The research focus on dandelion has progressively broadened to encompass gastric cancer, colorectal cancer, breast cancer, and various other malignant tumors [19, 20]. Research on the aforementioned two dandelion extracts has been relatively extensive, while studies on DF are relatively rare in clinical practice. Flavonoids are widely found in a diverse range of natural plants, exhibiting intricate structures and a wide array of variations. Multiple studies have suggested that flavonoids possess a wide spectrum of pharmacological functions, including scavenging free radicals, regulating the cardiovascular and cerebrovascular system, reducing blood glucose levels, exerting antioxidant effects, anti-tumor properties, anti-inflammatory and analgesic effects, and improving immunity. With their complex and diverse structures, multiple action sites, and low toxicity, these compounds have the potential to be further explored for the development of new drugs [21]. Research on the impact of DF on esophageal squamous cell carcinoma revealed that the alcohol extract of DF efficiently suppressed the proliferation of esophageal squamous cell carcinoma cells, and hindered their metastasis by targeting EMT-related proteins. These effects were shown to be dependent on both time and concentration gradients [22]. Given the limited amount of foundational research on the connection between DF and MM, the focus of this research is to explore the effect of DF on the migration and invasion capabilities of MM cells utilizing in vitro experiments. The aim is to delve into the associated mechanisms of regulating the metastatic potential of myeloma cells, consequently offering novel insights into the treatment of MM using TCM.

## Materials and methods

### MM cell lines

The human MM cell line U266 (iCell-h266) and human normal plasma cells were derived from the Cell Resource Center at the Shanghai Institute of Biological Sciences, Chinese Academy of Sciences.

### Cell culture

U266 cells were preserved in RPMI1640 medium (Thermofisher, 12633012) supplemented with 10% fetal bovine serum (Thermofisher, 26010074) and 1% penicillin–streptomycin (Thermofisher, 15140122) at 37 ℃ and 5% CO_2_ in a humidified atmosphere. Normal plasma cells were preserved in DMEM (Thermofisher, 12491015) medium with the same additives under the same conditions. Upon reaching 80%-90% confluence, the cells were dissociated using 0.25% trypsin (Thermofisher, 27250018) for passage.

### Preparation of DF

3-hydroxyflavone (Sigma, CAS No. 577-85-5) was dissolved in DMSO, filtered, and diluted in MEM to create a 1 mol/L stock solution, stored at 4 °C with DMSO concentration under 0.1%. Based on the IC50 of 253.6 µmol/L for DF, 250 µmol/L and 500 µmol/L solutions were prepared [23].

### Detection of colony forming ability

In a plate clone formation assay, U266 cells were trypsinized, plated at 500 cells/dish, and incubated at 37 °C with 5% CO_2_ for 14 days. Post-incubation, cells were washed, fixed, stained, and photographed for colony analysis.

### Cell proliferation activity detection

Two days post-transfection, MM cells were seeded in 96-well plates and cultured at 37 °C with 5% CO_2_ for 24 h. Then, 10 μL of CCK-8 solution (Sigma, 96992) was added to each well and incubated in darkness for 2 h before measuring the optical density at 450 nm using an automatic ELISA reader.

### Transwell and migration assay

Healthy MM cells were rinsed with PBS and cultured in MEM; controls in plain MEM and experimental groups in 250 µmol/L and 500 µmol/L DF for 24 h. After discarding the old medium and rinsing with PBS, cells were trypsinized, digestion stopped with MEM containing 10 g/L BSA, and cells were resuspended. For invasion and migration assays, Transwell chambers with and without Matrigel coating were used. A 200 µL cell suspension was placed in the upper chamber, and 500 µL of complete medium in the lower chamber, ensuring no bubbles formed. After incubating for 24 h, chambers were rinsed, fixed with methanol, and stained with diluted crystal violet. Cells were quantified using a light microscope at 100× magnification for migration and 200× for counting, with results averaged from five fields.2.7 Scratch experiment.

In a scratch assay, cells grown to near-confluence are scratched with a sterilized pipette tip, washed with PBS, and then incubated. Images are taken over time to monitor cell migration and scratch closure, with analysis focusing on the rate of closure and migration percentage at each time point.

### Calculation of cell apoptosis rate

After transfecting MM cells for 2 days, they were harvested, rinsed twice with PBS. Modified the density to 1 × 106/mL. Subsequently, a blend of 5 μL of PI and 5 μL of Annexin V/FITC (abcam, ab14085) was introduced into the cell suspension (400 μL). The cells were then cultured in darkness for 25 min. The apoptosis rate was immediately measured by BD-LSR-II flow cytometry.

### Real-time quantitative PCR

Total RNA was isolated from MM cells in the logarithmic growth phase with TRIZOL reagent.

**Table:** 

Genes	Forward primers	Reverse primers
mArg1	TTTTTCCAGCAGACCAGCTT	AGAGATTATCGGAGCGCCTT
MMP-2	CTCAGATCCGTGGTGAGATCT	CTTTGGTTCTCCAGCTTCAGG
MMP-9	ATCCAGTTTGGTGTCGCGGAGC	GAAGGGGAAGACGCACAGCT
TIMP-1	TTCCGACCTCGTCATCAGGG	ATTCAGGCTATCTGGGACCGC
TIMP-2	TGGAAACGACATTTATGGCAACC	ACAGGAGCCGTCACTTCTCTTGAT
β-actin	TCATGAAGTGTGACGTTGACATCCT	CCTAGAAGCATTTGCGGTGCACGATG
NOS	CAGCGGGATGACTTTCCAAG	AGGCAAGATTTGGACCTGCA
caspase-3	CCTCAGAGAGACATTCATG	GCAGTAGTCGCCTCTGAAGGA
caspase-9	AGTTCCCGGGTGCTGTCTAT	GCCATGGTCTTTCTGCTCAC
Bax	AGCAAACTGGTGCTCAAGGC	CCACAAAGATGGTCACTGTC
Bcl-2	GTGGTGGAGGAACTCTTCAG	GTTCCACAAAGGCATCCCAG

### Western blot

Western blot was employed to analyze proteins. The total proteins were extracted from the cell lysates of each group after washing with PBS. The total proteins in each set were isolated through vertical electrophoresis using SDS polyacrylamide gel, and then moved to the membrane. The cells were placed with 5% skim milk at room temperature for an hour, treated with the appropriate primary antibody, washed in PBS containing 0.1% Tween-20, and exposed to the corresponding diluted secondary antibody at room temperature for an hour. The cells were then scanned and stored.

### Statistical analysis

SPSS 16.0 was employed for data analysis. The measurement data were reported as mean ± SD, and the homogeneity of variance was tested. The independent sample t-test was conducted to compare means between the two sets, while One-way ANOVA and F test were conducted for comparing means among multiple groups. Statistical significance was determined as P < 0.05.

## Results

### DFs inhibited the proliferation of MM cells

**Fig. 1:**
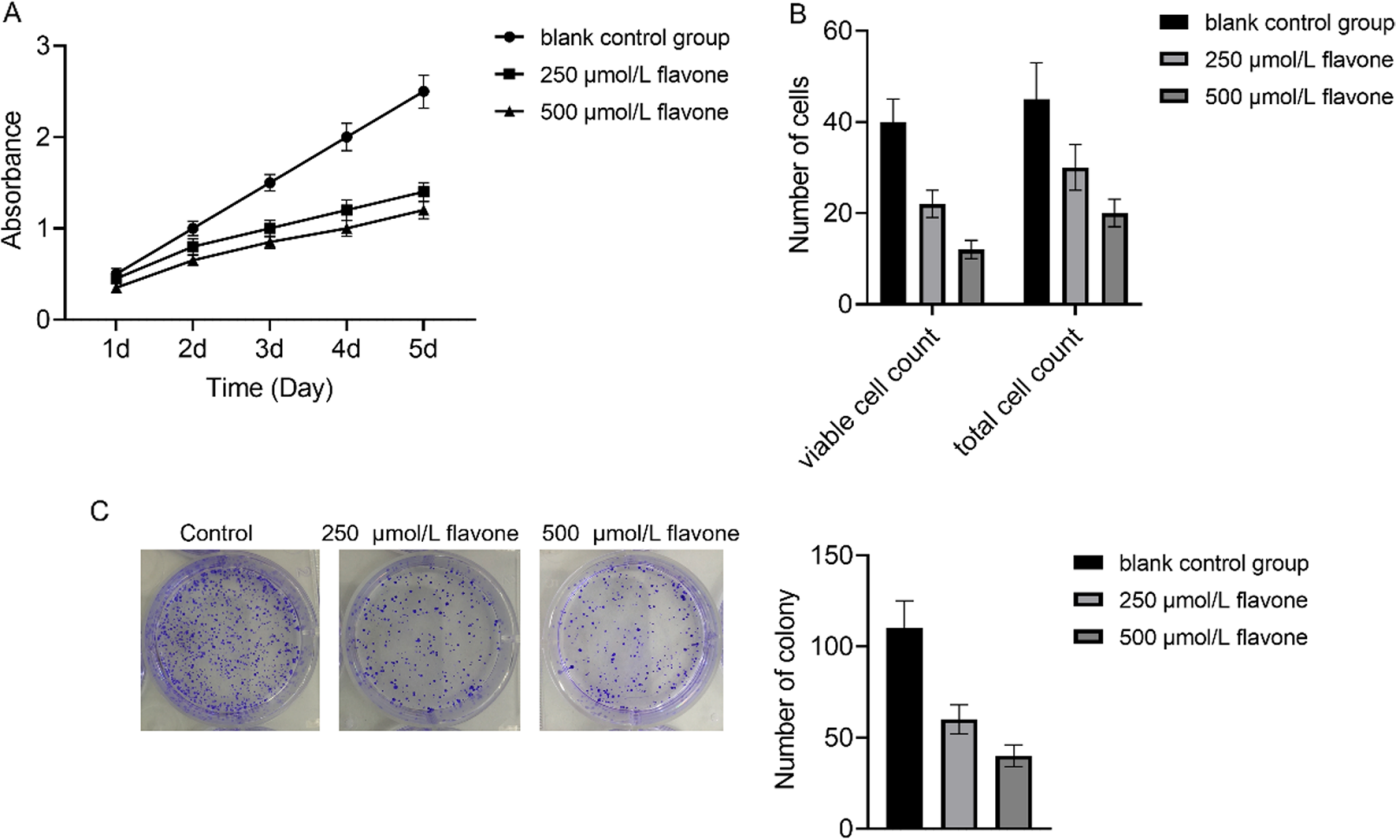
Inhibition of MM cell proliferation by DFs. **A** CCK-8 for cell proliferation detection. **B** Effect of MM cell proliferation. **C** Colony formation assay for colony-forming ability measurement

The impact of DF on MM cell proliferation was evaluated by the CCK-8 method. It was noted that with an increase in the concentration of DF, the rate of cell proliferation reduced. This difference from the control set was found to be dramatic, indicating that DF inhibited MM cell proliferation in a concentration-dependent manner (Fig. 1A). Furthermore, we assessed the impact of DF treatment for 10 days on the colony-forming capacity of MM cells through a colony formation assay. The findings revealed a marked decrease in the sum of MM cell colonies in the DF-treated group in comparison to the control group (Fig. 1C). We investigated the impact of DF on the growth of MM cell through cell counting analysis. Following treatment with varying concentrations of DF for 2 days, a gradual reduction in both viable cells and total cell count was observed with increasing drug concentration. These findings exhibited great differences contrast to the control group, indicating that DF suppressed the proliferation of MM cells in a concentration-dependent manner (Fig. 1B). The findings revealed that DF efficiently suppressed the proliferation of MM cells.

### DFs inhibited the migration and invasion of MM cells

**Fig. 2:**
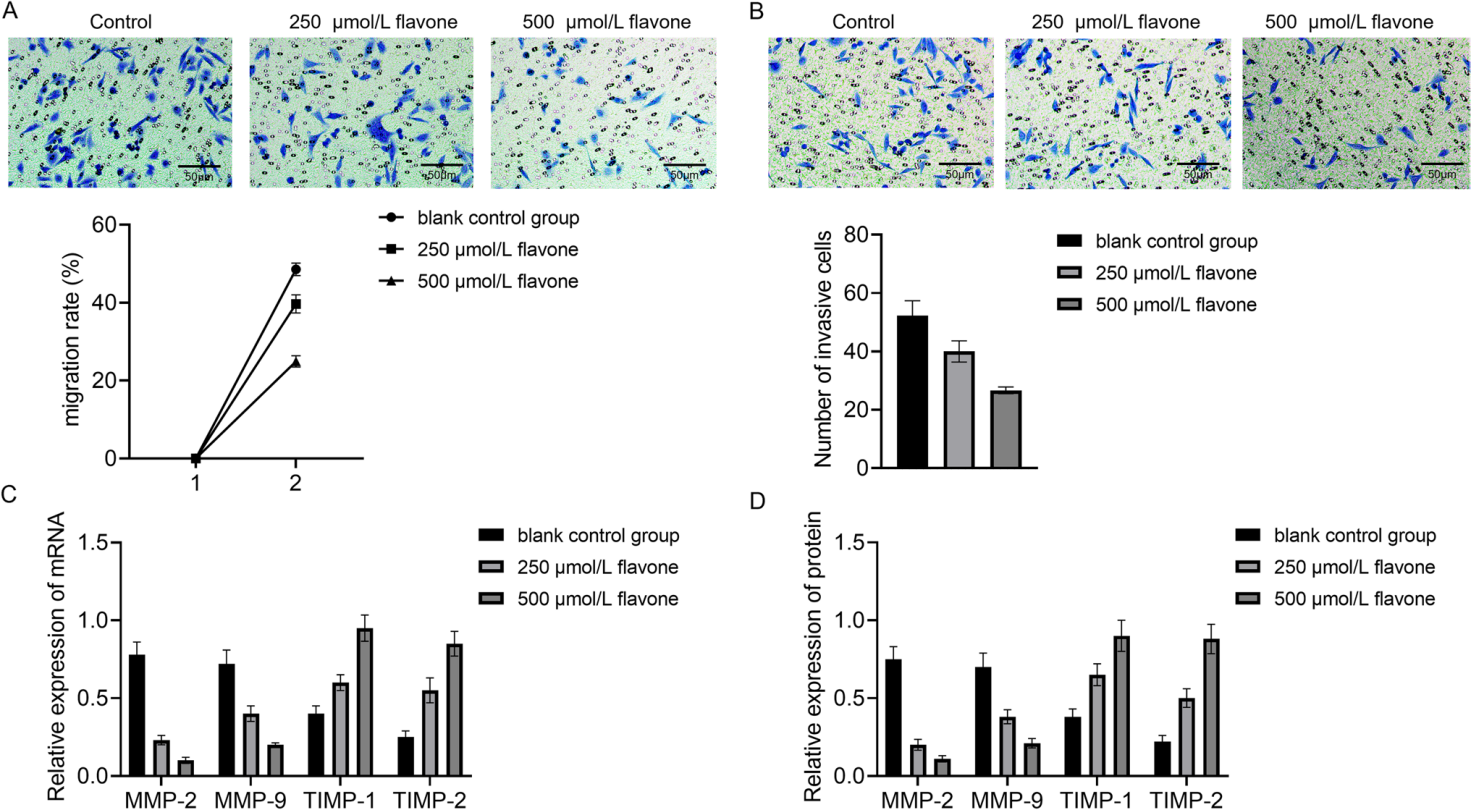
Inhibition of MM cell migration and invasion by DFs. **A** Comparison of migration ability of MM cells before and after DF treatment. **B** Effect of DFs. **C** RT-PCR and **D** ELISA for MMP-2, MMP-9, TIMP-1 and TIMP-2 mRNA expression detection in MM cells regulated by DF

A cell scratch assay was conducted on MM cells exposed to varying concentrations of DF to to assess cellular migration over a 24-h period. The scratch healing rates for the control set, the experimental set was subjected to 250 µmol/L of flavone, and the experimental set was subjected to 500 µmol/L of flavone were recorded as (48.58 ± 1.67) %, (39.68 ± 2.32) % and (24.91 ± 1.44) %. Statistically significant variances were observed among the mentioned sets, with significant variations between each pair of sets. The scratch healing rate of the experimental set treated with 250 µmol/L of flavonoid and the set treated with 500 µmol/L of flavonoid was notably decreased compared to that of the control set. Moreover, the scratch healing rate of 500 µmol/L flavonoid experimental set was notably decreased compared to that of 250 µmol/L flavonoid experimental set, with mathematically dramatic variations (Fig. 2A). Transwell assay was utilized to evaluate the impact of DF on the invasion capabilities of MM cells. A notable decrease within the cell count transiting across the membrane was observed in both the experimental groups treated with 250 µmol/L and 500 µmol/L of flavonoid, compared to the control group [(52.33 ± 5.03) vs (40.00 ± 3.61), (26.66 ± 1.15)] (Fig. 2B). Endogenous inhibitors of MMPs are tissue TIMPs that regulate MMPS activity. The equilibrium between MMPs and TIMPs often leads to dynamic changes in malignant tumors. MMP-9 and TIMP-1 are recognized for their cell-specific characteristics, and the equilibrium between MMP-9 and TIMP-1 expression in tumor cells is closely connected with the biological characteristics of tumors. The findings demonstrated that DF was capable of modulating the mRNA expression of MMP-2, MMP-9, TIMP-1 and TIMP-2 in MM cells. Compared to the control set without treatment, the flavonoid-treated set exhibited notably elevated relative expression levels of TIMP-1 and TIMP-2 mRNA, associated with great reductions in MMP-2 and MMP-9 mRNA, with mathematically dramatic differences between the sets (Fig. 2C). What’s more, the protein levels of MMP-2, MMP-9, TIMP-1 and TIMP-2 in MM cells were assessed by ELISA, yielding results consistent with the mRNA findings (Fig. 2D).

### DFs increased MM cell apoptosis

**Fig. 3:**
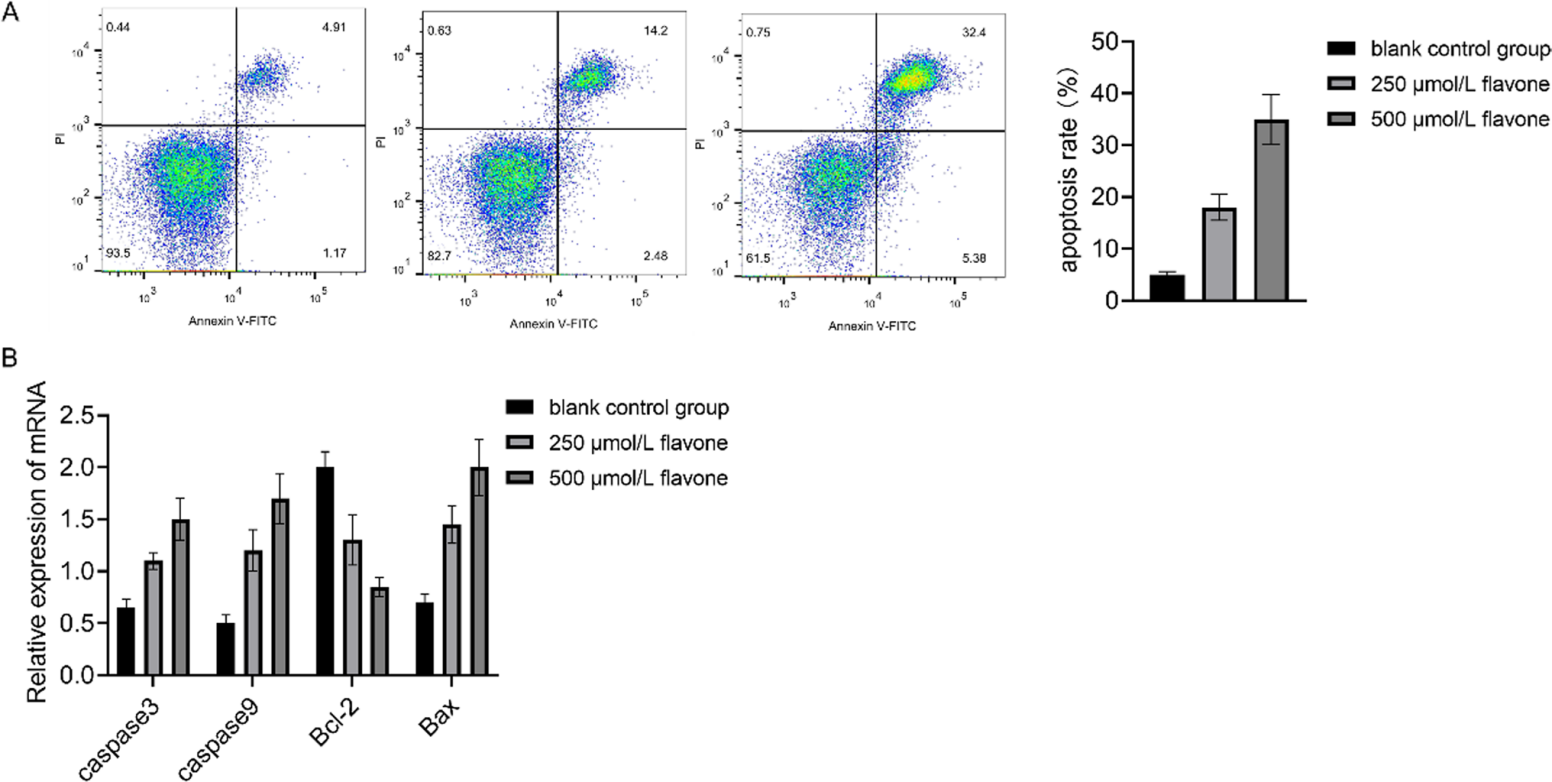
Increase of MM cell apoptosis by DFs. **A** Detection of apoptosis by flow cytometry. **B** Measurement of the expression of apoptosis-related molecules caspase3, caspase9, Bcl-2, and Bax by quantitative PCR

Flow cytometry analysis showed a concentration-dependent increase in the proportion of apoptosis in MM cells following treatment with DF (Fig. 3A). The Bcl family is a key molecular player influencing cell proliferation and apoptosis, pivotal in the onset and progression of numerous tumors. RT-PCR results demonstrated the regulatory impact of DF on the expression of Bax and Bcl-2 in MM cells. Compared to the control set, there was a notable enhancement in the relative expression of Bax and a marked decrease in Bcl-2 within the DF group (Fig. 3B).

### DFs inhibited MΦs cell polarization to M2-type MΦs

**Fig. 4:**
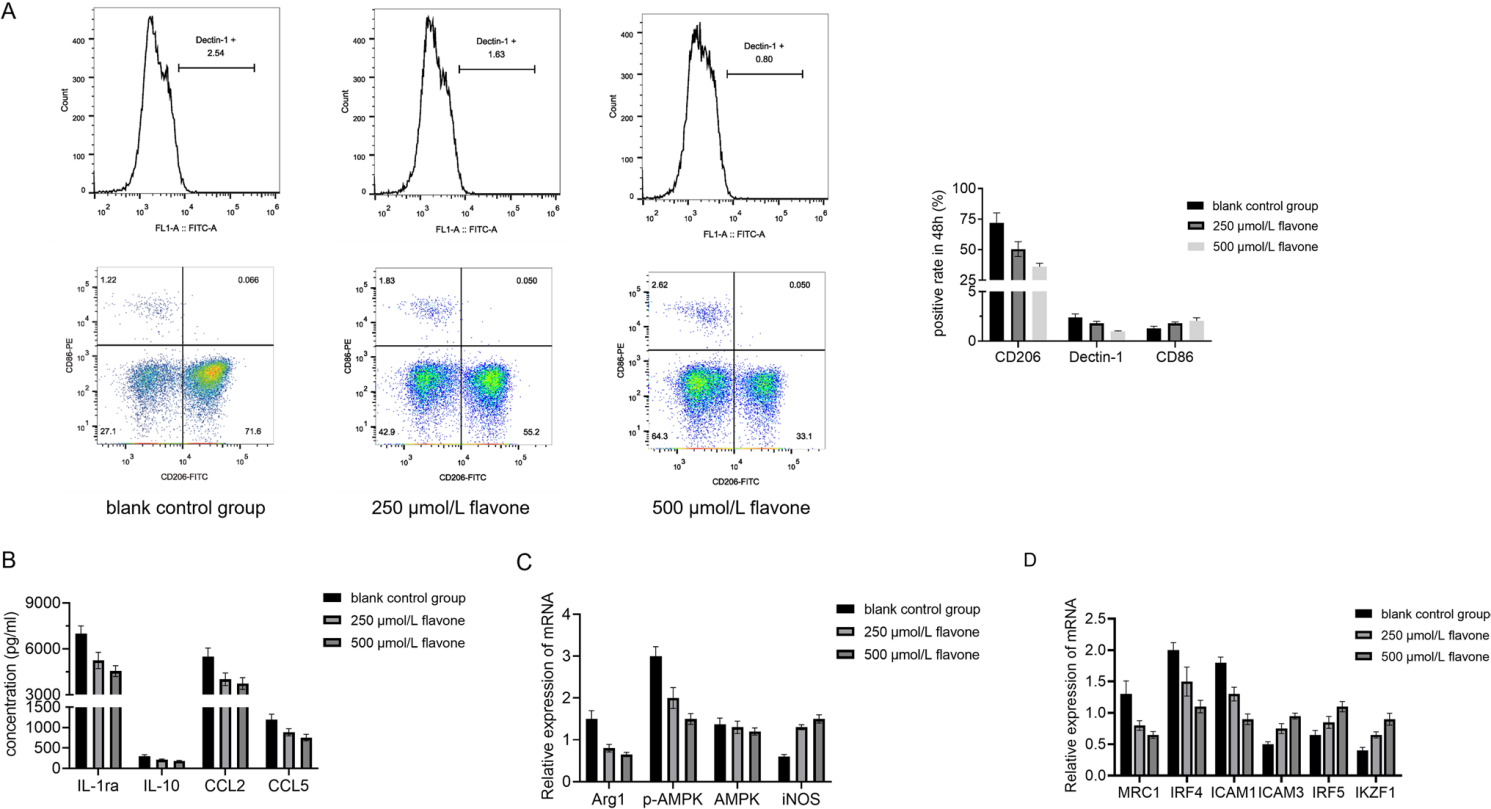
Inhibition of MΦs cell polarization to M2-type MΦs by DFs. **A** Detection of CD206 and Dectin-1 (CD369) on M2 macrophages and CD86 on M1 macrophages by flow cytometry. **B** ELISA analysis revealed that DF enhanced the secretion of M2 cytokines by macrophages, including IL-1ra, IL-10, CCL2, and CCL5. **C** Detection of the expression of Arg1 and p-AMPK in M2 macrophages and iNOS in M1 macrophages by RT-PCR. **D** Detection of the expression of MRC1, IRF4 and ICAM1 characteristic molecules of M2 type and ICAM3, IRF5 and IKZF1 characteristic molecules of M1 type by qRT-PCR

Following 24-h stimulation of MΦs with DF, flow cytometry indicated a decrease in the levels of M2 surface markers CD206 and Dectin-1 (CD369), alongside an increase in the level of M1 surface marker CD86 (Fig. 4A). ELISA analysis revealed that DF concentration-dependently inhibited the release of M2-type cytokines like IL-1ra, IL-10, CCL2 and CCL5 (Fig. 4B). qRT-PCR analysis revealed a decrease in the expression of Arg1 and p-AMPK, characteristic metabolic enzymes of M2 type, and an increase in the expression of iNOS, a characteristic metabolic enzyme of M1 type, upon DF stimulation of MΦs (Fig. 4C). Furthermore, qRT-PCR demonstrated a decrease in the levels of M2 characteristic molecules MRC1, IRF4 and ICAM1, coupled with an increase in the levels of M1 characteristic molecules ICAM3, IRF5 and IKZF1. The above findings collectively indicated that DF could impede the polarization of MΦs to M2 (Fig. 4D).

### DFs inhibited the expression of PI3K/AKT signaling pathway related proteins in MM cells

**Fig. 5:**
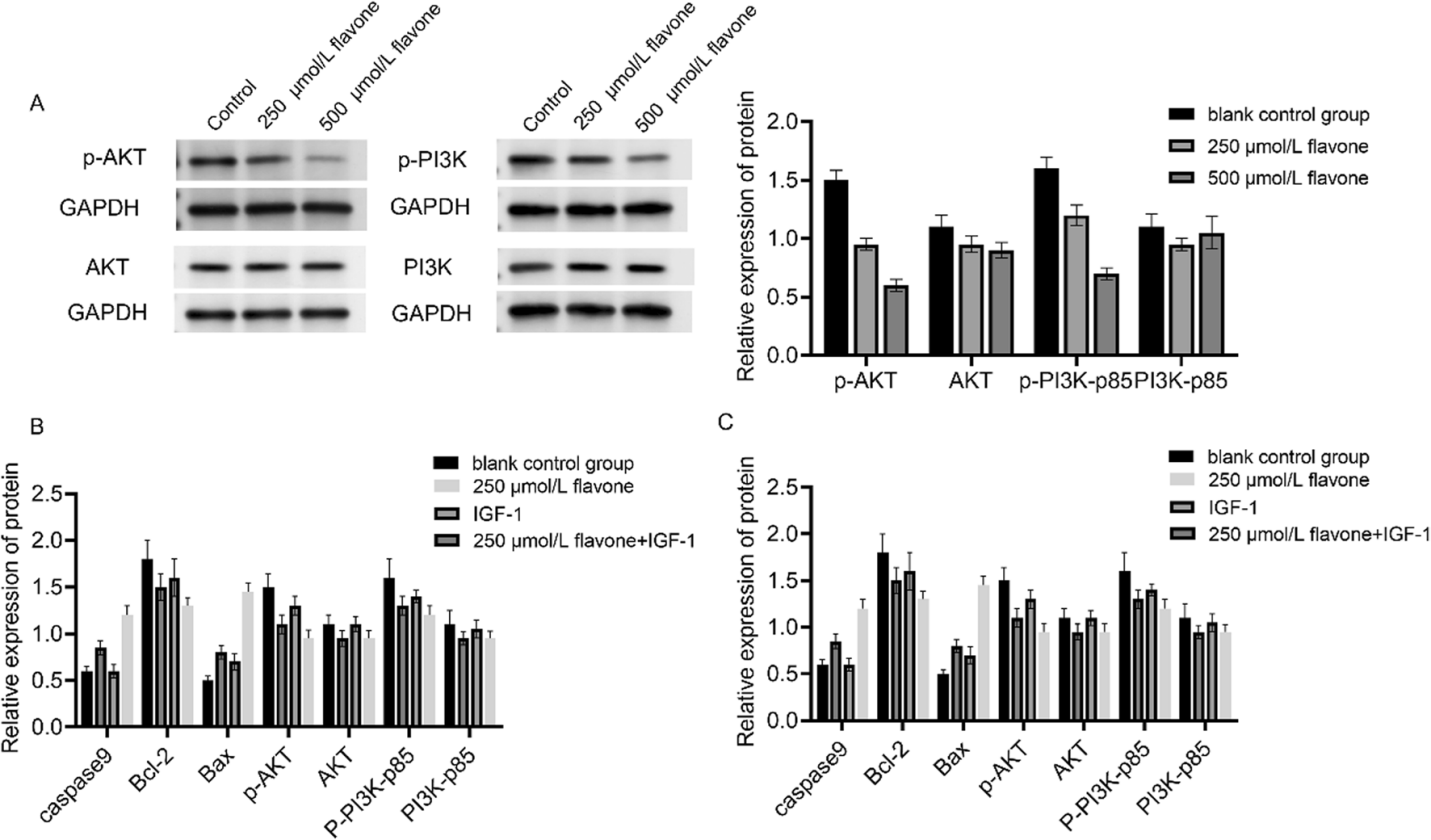
Inhibition of the expression of proteins involved in PI3K/AKT/mTOR signaling pathway in MM cells by DFs. **A** Detection of the protein expression levels of AKT, p-AKT, PI3K-p85 and p-PI3K-p85 in MM cells by western blot. **B** Detection of the DFs effectiveness on apoptosis proteins and signaling pathway proteins in MM cells after the activation of PI3K/AKT pathway by IGF-I by ELISA assay. **C** Detection of the effectiveness of DFs on apoptosis proteins and signaling pathway proteins in MM cells after adding LY294002 to inhibit PI3K/AKT pathway by ELISA assay

To investigate the potential anti-MM effects of DFs targeting the PI3K/AKT signaling pathway, we assessed the activity of PI3K/AKT pathway in MM cells treated with DFs for 24 h using western blot analysis. The expression of proteins associated with p-PI3K and p-AKT pathways was inhibited in a concentration dependent manner in MM cells dealt with DF for 24 h compared to the control set (Fig. 5A). This suggested that DF can suppress the PI3K/AKT signaling pathway. In addition, to investigate the impact of DF-induced inhibition of the PI3K/AKT signaling pathway on MM cell apoptosis, MM cells were divided into four groups: Con group, DF group, IGF-1 group and DF + IGF-1 group. Cells in IGF-1 group and DF + IGF-1 group were pretreated with IGF-1 (final concentration of 100 ng/ml) for 1 h, and then treated with DF for 24 h, respectively. The activation of the PI3K/AKT pathway by IGF-1 partially reduced the protein expression of Bax and Cleaved caspase-9 induced by DF, as well as the protein expression of Bcl-2 inhibited by DF in MM cells (Fig. 5B). Conversely, upon adding the PI3K inhibitor LY294002 (RPMI8226 IOμM, MM.IS 5 μM), an opposite result was observed (Fig. 5C).

## Discussion

Currently, MM remains incurable. Despite the ongoing approval of new drugs leading to great improvements in the overall survival of MM patients. They continue to face the challenges of recurrence and refractory. The growing focus on TCM has attracted an increasing number of scholars to explore its clinical applications, bringing new perspectives to the traditional treatment of tumors. Considering the current treatment landscape of MM and the theoretical framework outlined above, our research group carried out an experiment examining the impact of DF intervention on MM cells. The study sought to investigate the effectiveness of DF on MM cell proliferation, migration and apoptosis, while also exploring the involvement of the PI3K/AKT/mTOR pathway.

Dandelion can extract dandelion sterols, dandelion alcohol, flavonoids, polysaccharides and other components. In recent years, the research on its components has been increasing. Domestic and foreign studies have shown that dandelion has important effects, including its ability to inhibit tumor cell proliferation, guide tumor cell apoptosis, improve immunity and anti-inflammation. The research scope of dandelion has gradually expanded to include gastric cancer, colorectal cancer, breast cancer and other malignant tumors [19, 20]. Dandelion sterol is a pentacyclic triterpenoid compound with various biological activities. It can inhibit the production of inflammatory mediators, regulate the activities of inflammation-related and oxidation-related enzymes and related signaling pathways, and down-regulate the expression of tumor proliferation-related elements like Bax, Bcl-2 and cyclinD1, thereby demonstrating an anti-tumor effect [24]. Dandelion polysaccharide is also an important part of the pharmacological effects of dandelion, and its physiological effects are mainly manifested in antibacterial and anti-inflammatory, immunity enhancing, anti-oxidation, liver protection and anti-tumor [25]. In our recent research conducted, it was found that dandelion flavone (DF) effectively suppresses the proliferation of multiple myeloma (MM) cells in a manner that is influenced by both the duration of treatment and the concentration of the drug. Additionally, DF was observed to significantly limit the migration and invasion capabilities of MM cells. Importantly, this inhibitory effect on cell migration and invasion was found to intensify as the dosage of DF was increased. This suggests a dose-dependent relationship in the efficacy of DF in controlling these critical aspects of MM cell behavior.

TIMPs play a crucial role in impeding tumor advancement by suppressing the invasion and migration of tumor cells. Among the TIMP family members, TIMP-1 has been extensively investigated in many diseases due to its direct inhibitor of MMP-9 and its prominent expression in malignant tumors. Despite the heightened release of MMPs, this alone is inadequate to counteract the anti-migratory effect of TIMP expression. By working in conjunction, TIMP-1 and TIMP-2 effectively diminish the migration and invasion of MSCS [26]. In the current study, the expression of MMP-2 and MMP-9 mRNA in MM cells decreased with the increase of flavonoids concentration, while the expression of TIMP-1 and TIMP-2 mRNA increased with the increase of flavonoids concentration. DF could significantly activate the expression of pro-apoptotic proteins caspase3, caspase9 and Bax.

M2 Mφs are often induced in response to IL-4 and/or IL-13 (produced during the Th2 polarization of T lymphocytes) and highly express Arginase (Arg1), chitin enzymes such as Ym1, resistin-like molecule a (Fizzl), fatty acid translocases CD36 and CD206. Low expression of IL-12 and iNOS often occurs in a variety of pathological environments, such as viral intracellular infection, allergy, diabetes, etc. [27]. The immunosuppressive function of M2 Mφs makes antigens persist in vivo and forms chronic inflammation, which is closely related to tumorigenesis [28, 29]. The polarized tumors of Mφs exhibit similarities, and the Mφs infiltrated in the BM of MM patients are primarily M2 type [30]. It has been found that the polarization of Mφs is a dynamic reversible process, for example, inhibition of Colony stimulatingfactor-1 receptor (CSF-1R) on the surface of Mφs can repolarize M2 Mφs into M1 Mφs in the tumor microenvironment and exert tumor killing function [31]. Together, DF has been shown to effectively inhibit the M2 polarization of macrophages, which is significant in reducing the immunosuppressive environment that facilitates chronic inflammation and tumorigenesis. Our results described a decrease in the levels of M2 characteristic molecules MRC1, IRF4 and ICAM1, coupled with an increase in the levels of M1 characteristic molecules ICAM3, IRF5 and IKZF1. Besides, a decrease in the expression of Arg1 and p-AMPK, characteristic metabolic enzymes of M2 type, and an increase in the expression of iNOS, a characteristic metabolic enzyme of M1 type, upon DF stimulation of MΦs were found in our study. The PI3K/AKT/mTOR pathway is crucial to MM cell survival. PI3K activates AKT, and then activates its downstream mTOR and FOXO proteins, thereby regulating the proliferation of MM cells and anti-apoptosis of MM cells. It is understood that the PI3K/AKT pathway has a dramatic effect on the expression of IGF-1, IL-6 and other factors in MM cells, and the downstream target genes of PI3K/AKT pathway, such as mTOR and p53, are also closely associated with the pathogenesis of MM [32]. Targeted therapy for this pathway mainly includes strategies such as inhibition of PI3K or AKT and repair of PTEN [32]. Inhibition of this signaling pathway has emerged as a focal point for tumor prevention, targeted therapy and reversal of drug resistance. Researches have reported that silibinin can suppress the proliferation of MM cells and promote their apoptosis, and its possible molecular mechanism is connected with the restriction of PI3K/AKT/mTOR pathway expression [33]. Moreover, in vitro experimental studies have shown that polydatin can suppress the proliferation of myeloma cell RPMI8226 by inhibiting the phosphorylation of mTOR and p70s6k, and activate autophagy and induce apoptosis [34]. The study demonstrated that dandelion flavone (DF) is capable of inhibiting the phosphorylation of key signaling proteins, PI3K and AKT, which are crucial in the proliferation and survival pathways of multiple myeloma (MM) cells. This inhibition by DF leads to a suppression of MM cell growth. When recombinant human IGF-1, a growth factor known to activate the PI3K/AKT pathway, was introduced, it was observed that the inhibitory effects of DF on the MM cells were diminished, suggesting that IGF-1 can counteract the action of DF. Conversely, when the PI3K inhibitor LY294002, which specifically blocks the PI3K pathway, was added to the experimental setup, it intensified the suppressive effects of DF on MM cells. This contrasting response further confirms the critical role of the PI3K/AKT pathway in mediating the effects of DF and underscores the potential of combining DF with specific pathway inhibitors to enhance therapeutic outcomes in MM treatment.

Taken together, the present study found that taraxacine flavonoids inhibited the proliferation, migration and invasion of MM cells and induced apoptosis of MM cells by targeting the PI3K/AKT pathway, which may potentially offer a novel theoretical foundation for the clinical management of MM. This study was limited to in vitro experiments using one or a few cell lines and did not involve a broader range of cell types or in vivo models, which limits the general applicability of the results. There is a lack of data on the long-term therapeutic effects and safety of DF, which are crucial for clinical application. Although the study explored the regulation of the PI3K/AKT pathway, other important signaling pathways and molecular mechanisms may not have been addressed. Further, we will use different MM cell lines and establish animal models to research and verify the anti-tumor activity and mechanisms of DF in more complex biological systems. Exploring the mechanisms of DF's effects on MM cells, including whether it affects other biomarkers and signaling pathways, and how these pathways interact.

### Supplementary Information

Supplementary Material 1.

## Data Availability

Data from this study have been stored in the figshare online database, https://figshare.com/s/c9a9907053860f89c0da.
