# Supplementary material for: Anti-tumor effect of dandelion flavone on multiple myeloma cells and its mechanism
Source: Discov Oncol. 2024 Jun 8;15:215. doi: 10.1007/s12672-024-01076-z (PMC11162407; doi:10.1007/s12672-024-01076-z)
Supplement: Supplementary file 1 — Supplementary Material 1. [file 12672_2024_1076_MOESM1_ESM.pptx]

## Slide 1
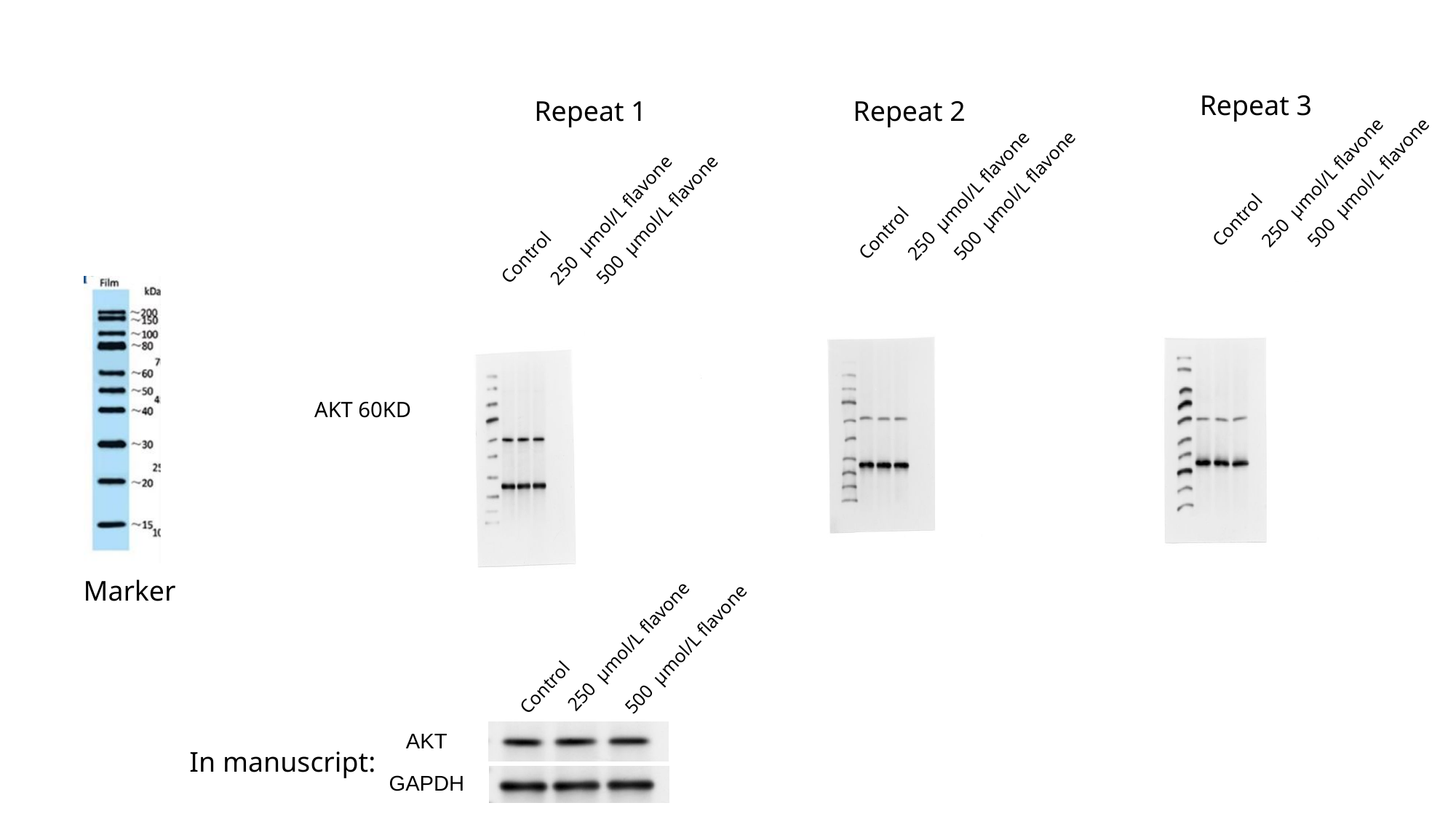

Repeat 3
Repeat 1
Repeat 2
250 μmol/L flavone
500 μmol/L flavone
250 μmol/L flavone
500 μmol/L flavone
Control
250 μmol/L flavone
500 μmol/L flavone
Control
Control
AKT 60KD
Marker
250 μmol/L flavone
500 μmol/L flavone
Control
| AKT |
| --- |
In manuscript:
| GAPDH |
| --- |

## Slide 2
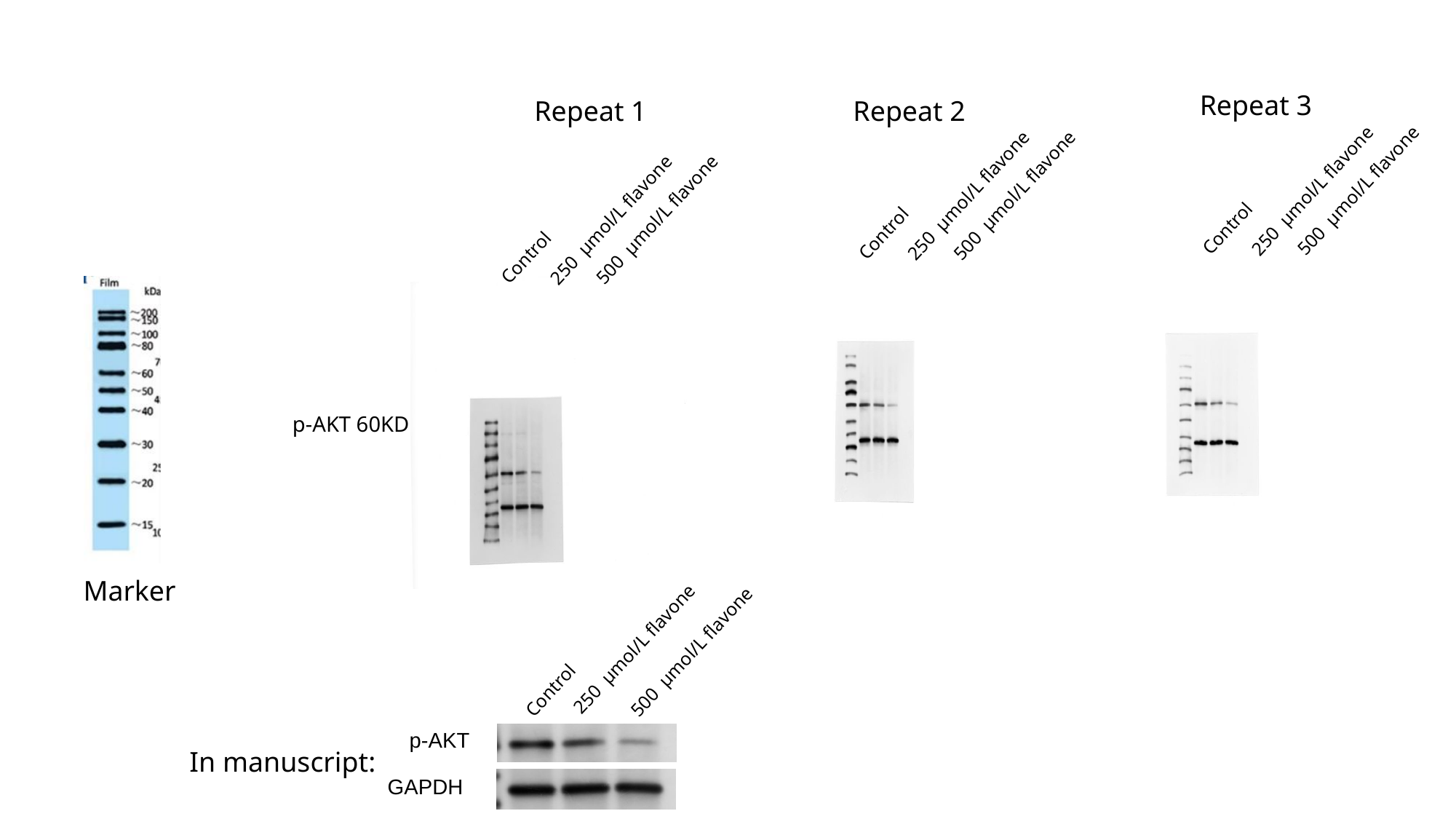

Repeat 3
Repeat 1
Repeat 2
250 μmol/L flavone
250 μmol/L flavone
500 μmol/L flavone
500 μmol/L flavone
250 μmol/L flavone
Control
500 μmol/L flavone
Control
Control
p-AKT 60KD
Marker
250 μmol/L flavone
500 μmol/L flavone
Control
| p-AKT |
| --- |
In manuscript:
| GAPDH |
| --- |

## Slide 3
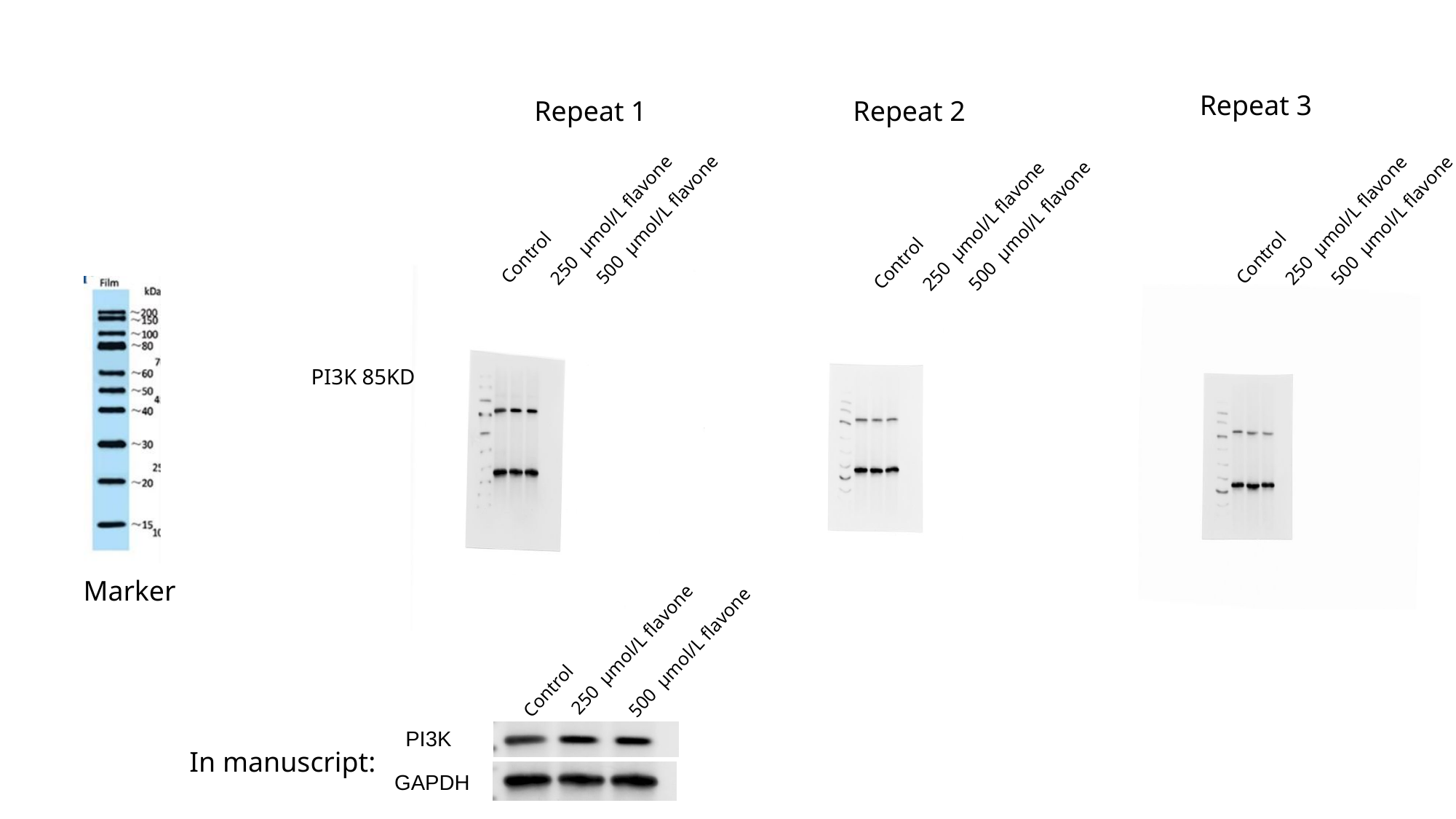

Repeat 3
Repeat 1
Repeat 2
250 μmol/L flavone
250 μmol/L flavone
250 μmol/L flavone
500 μmol/L flavone
500 μmol/L flavone
500 μmol/L flavone
Control
Control
Control
PI3K 85KD
Marker
250 μmol/L flavone
500 μmol/L flavone
Control
| PI3K |
| --- |
In manuscript:
| GAPDH |
| --- |

## Slide 4
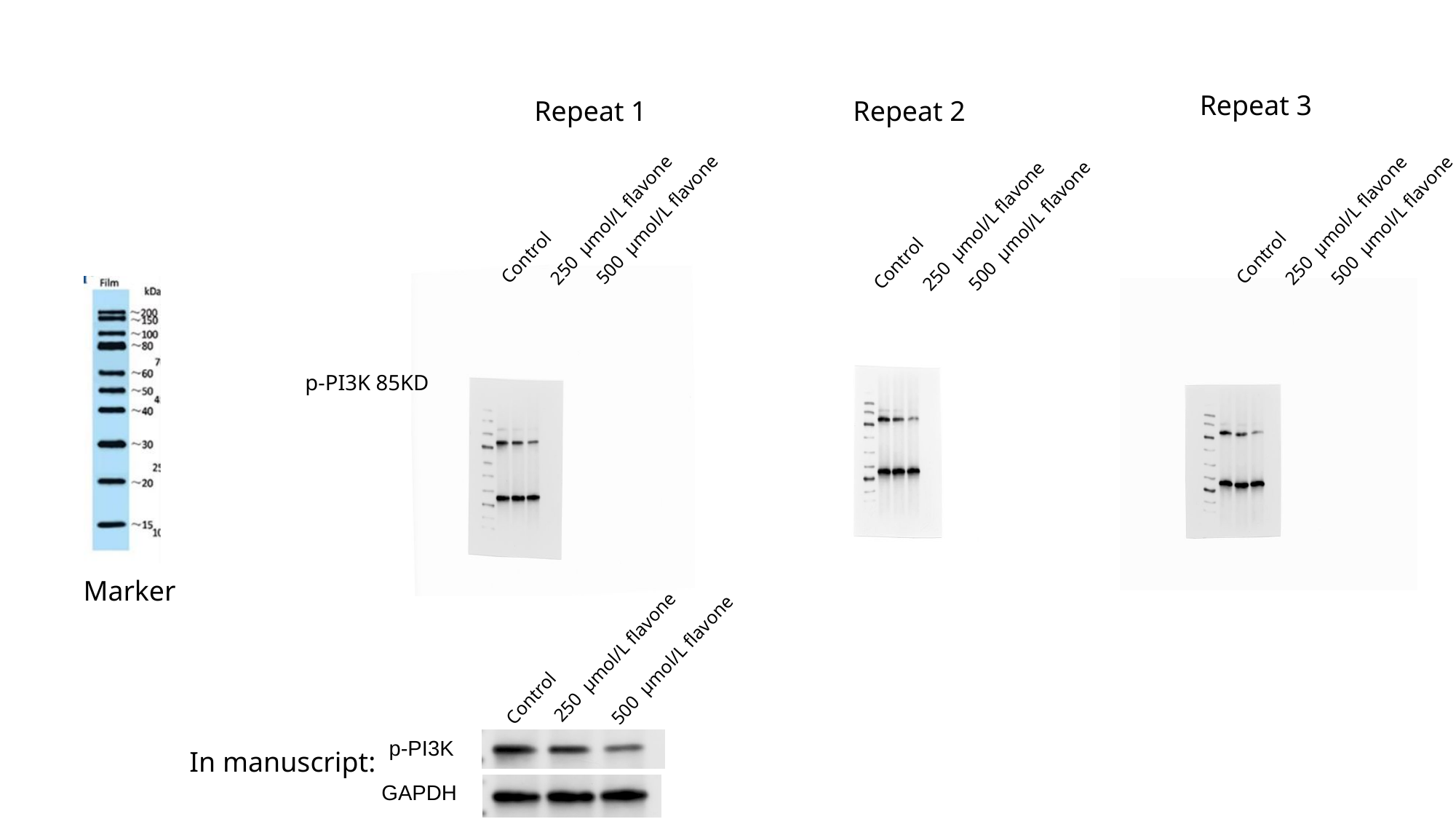

Repeat 3
Repeat 1
Repeat 2
250 μmol/L flavone
250 μmol/L flavone
250 μmol/L flavone
500 μmol/L flavone
500 μmol/L flavone
500 μmol/L flavone
Control
Control
Control
p-PI3K 85KD
Marker
250 μmol/L flavone
500 μmol/L flavone
Control
| p-PI3K |
| --- |
In manuscript:
| GAPDH |
| --- |
